# Effects of IL-6, JAK, TNF inhibitors, and CTLA4-Ig on knee symptoms in patients with rheumatoid arthritis

**DOI:** 10.1038/s41598-024-66064-3

**Published:** 2024-07-02

**Authors:** Koichi Murata, Ryuji Uozumi, Takayuki Fujii, Akira Onishi, Kosaku Murakami, Hideo Onizawa, Masao Tanaka, Akio Morinobu, Shuichi Matsuda

**Affiliations:** 1 Department of Advanced Medicine for Rheumatic Diseases, Kyoto University Graduate School of Medicine, 54 Shogoin-Kawahara-cho, Sakyo, Kyoto 606-8507 Japan; 2 Department of Orthopaedic Surgery, Kyoto University Graduate School of Medicine, Sakyo, Kyoto Japan; 3 Department of Industrial Engineering and Economics, Tokyo Institute of Technology, Tokyo, 152-8552 Japan; 4 Division of Clinical Immunology and Cancer Immunotherapy, Center for Cancer Immunotherapy and Immunobiology, Kyoto University Graduate School of Medicine, Sakyo, Kyoto 606-8501 Japan; 5 Department of Rheumatology and Clinical Immunology, Kyoto University Graduate School of Medicine, Sakyo, Kyoto 606-8507 Japan

**Keywords:** Rheumatoid arthritis, Knee arthritis, Disease-modifying antirheumatic drugs, CTLA4-Ig, IL-6, Rheumatology, Rheumatic diseases, Rheumatoid arthritis

## Abstract

This study aims to identify factors influencing the alleviation of knee joint symptoms in patients with rheumatoid arthritis treated with biologic or target synthetic disease-modifying antirheumatic drugs (b/tsDMARDs). Among 2321 patients who started b/tsDMARDs between 2010 and 2023, we focused on 295 patients who had knee swelling or tenderness at the initiation of b/tsDMARDs and continued b/tsDMARDs at least 3 months, with recorded knee symptoms 6 months later. Symptom relief after 6 months was 78.2% for interleukin 6 (IL-6) inhibitors, 68.6% for Janus kinase (JAK) inhibitors, 65.8% for tumor necrosis factor (TNF) inhibitors, and 57.6% for cytotoxic T lymphocyte-associated antigen-4-Ig (CTLA4-Ig). The initial use of b/tsDMARDs and the use of IL-6 inhibitors in comparison to CTLA4-Ig emerged as a significant factor associated with the improvement of knee joint symptoms. Among 141 patients who underwent knee radiography at baseline and two years later, the deterioration in knee joint radiographs was 7.7% for IL-6 inhibitors, 6.3% for JAK inhibitors, 21.9% for TNF inhibitors, and 25.9% for CTLA4-Ig. The use of IL-6 inhibitors was a significant factor associated with the improvement of knee joint symptoms and the inhibition of joint destruction compared to CTLA4-Ig.

## Introduction

Rheumatoid arthritis (RA) is a multifactorial autoimmune disease of unknown etiology with an estimated prevalence of 0.5 to 1% in adults, which can damage joints and deteriorate the quality of life^1^. Recent advances in treatment strategies using biological disease-modifying antirheumatic drugs (bDMARDs) and targeted synthetic disease-modifying antirheumatic drugs (tsDMARDs) improve disease activity and quality of life^2,3^. However, limited information is available regarding large weight-bearing joints.

The weight-bearing joints of the lower limbs are important for daily activities^4^. Damage to the weight-bearing joints results in substantial deterioration of both daily activities and quality of life. The suppressive effect of biological/targeted synthetic disease-modifying antirheumatic drugs (b/tsDMARDs) on the progression of joint structural damage has been evaluated using the modified Sharp-van der Heijde scoring method^5^, which assesses small joints of the hands and feet. Although the method helps estimate the treatment efficacy early in the disease process, it does not provide any information about damage to large joints.

The knees are the body’s largest weight-bearing joint, with a joint surface 26-fold greater than that of the metacarpophalangeal joint^6^. A large volume of synovial tissue may require large amounts of drug. In addition, early suppression of synovitis may be necessary. Previous reports indicate that tumor necrosis factor (TNF) inhibitors were ineffective in inhibiting the progression of joint destruction once it reached a degree beyond mild destruction in weight-bearing joints^7^. These findings suggest that different strategies are required to treat knee symptoms in patients with RA.

This study investigates the factors influencing improvements in knee symptoms who started b/tsDMARDs. We also analyze the risk factors for structural damage to the knee joint via radiography.

## Methods

### Patients

The analyses in the current study were conducted using the Kyoto University Rheumatoid Arthritis Management Alliance (KURAMA) cohort database, with approval from the ethics committee of Kyoto University Hospital. Written informed consent was obtained from all patients. The KURAMA cohort was established at Kyoto University Hospital in 2010 to strictly control RA using patients’ clinical and laboratory data for clinical investigations^8–10^. Clinical, biological, and functional data were recorded for each patient at the initial visit and at every subsequent visit, which enabled the assessment of the occurrence, resolution, and treatment of knee symptoms in RA. All patients with RA satisfied the classification criteria of the American College of Rheumatology (ACR) criteria revised in 1987^11^ or the ACR/European League Against Rheumatism (EULAR) criteria revised in 2010^12^. Owing to the low sensitivity of the 2010 ACR/EULAR classification criteria for seronegative patients, we used the 1987 and 2010 ACR/EULAR classifications.

Patients who started b/tsDMARDs between January 2010 and August 2023 (*n* = 2321) were retrospectively analyzed. The baseline was defined as the time at which patients started using the b/tsDMARDs. Patients with swelling or tenderness in the knee joint at baseline (*n* = 573) were included. A total of 438 patients returned for follow-up 6 months later with available medical record. Patients with a history of total knee arthroplasty (*n* = 18) or who had undergone total knee arthroplasty on the symptomatic side during follow-up (*n* = 7) were excluded.

The following b/tsDMARDs were used: infliximab (IFX), adalimumab (ADA), golimumab (GLM), etanercept (ETN), certolizumab, pegol (CTZ), abatacept (ABT), tocilizumab (TCZ), sarilumab (SAR), tofacitinib (TOF), baricitinib (BAR), peficitinib (PEF), and filgotinib (FIL). The medications were categorized into four groups based on their mode of action^13^: TNF inhibitors (IFX, ADA, GLM, ETN, CTZ), T-cell costimulation inhibitor (cytotoxic T lymphocyte-associated antigen-4-Ig [CTLA4-Ig] or ABT), interleukin 6 (IL-6) inhibitors (TCZ, SAR), and Janus kinase (JAK) inhibitors (TOF, BAR, PEF, FIL).

### Radiographic evaluation

The knee joints were evaluated using the Larsen grade^14^ by two researchers trained in radiographic assessments (K.M. and T.F.). Disagreements were resolved by consensus. The Larsen grade was defined as follows: grade 0 (no change), the joint was normal in appearance; grade I (slight changes), periarticular soft tissue swelling, osteoporosis, and slight narrowing of the joint spaces; grade II (definite early changes), narrowing of the joint spaces and erosion of all except the weight-bearing joints; grade III (moderate destructive changes), narrowing of the joint spaces and erosion of all joints; grade IV (severe destructive changes), extreme joint space narrowing, erosion of all joints, and bone deformation at the weight-bearing joints; grade V (mutilating changes), disappearance of the original articular surfaces and gross bone deformation. Aggravations other than Larsen grades 0–I were recorded^4^.

### Statistical analysis

All statistical analyses were conducted using SAS statistical software, version 9.4, and JMP Pro 15 software (SAS Institute Inc., Cary, NC, USA). The Kruskal–Wallis test and one-way analysis of variance (one-way ANOVA) were used to compare numerical data across three or more groups. The Steel–Dwass test was used to test for differences in disease activity. Pearson’s chi-square test was used to analyze categorical variables. The Cochran–Armitage test was used to assess the trend of the alleviation of knee joint symptoms across the group. A multivariate logistic regression analysis was conducted to evaluate the risk factors associated with improving knee joint symptoms.

### Ethical approval

This study was conducted following the Declaration of Helsinki and approved by the Ethics Committee of Kyoto University Hospital (E1308). Written informed consent was obtained from all participants.

## Results

### Factors contributing to the alleviation of knee symptoms

**Figure 1:**
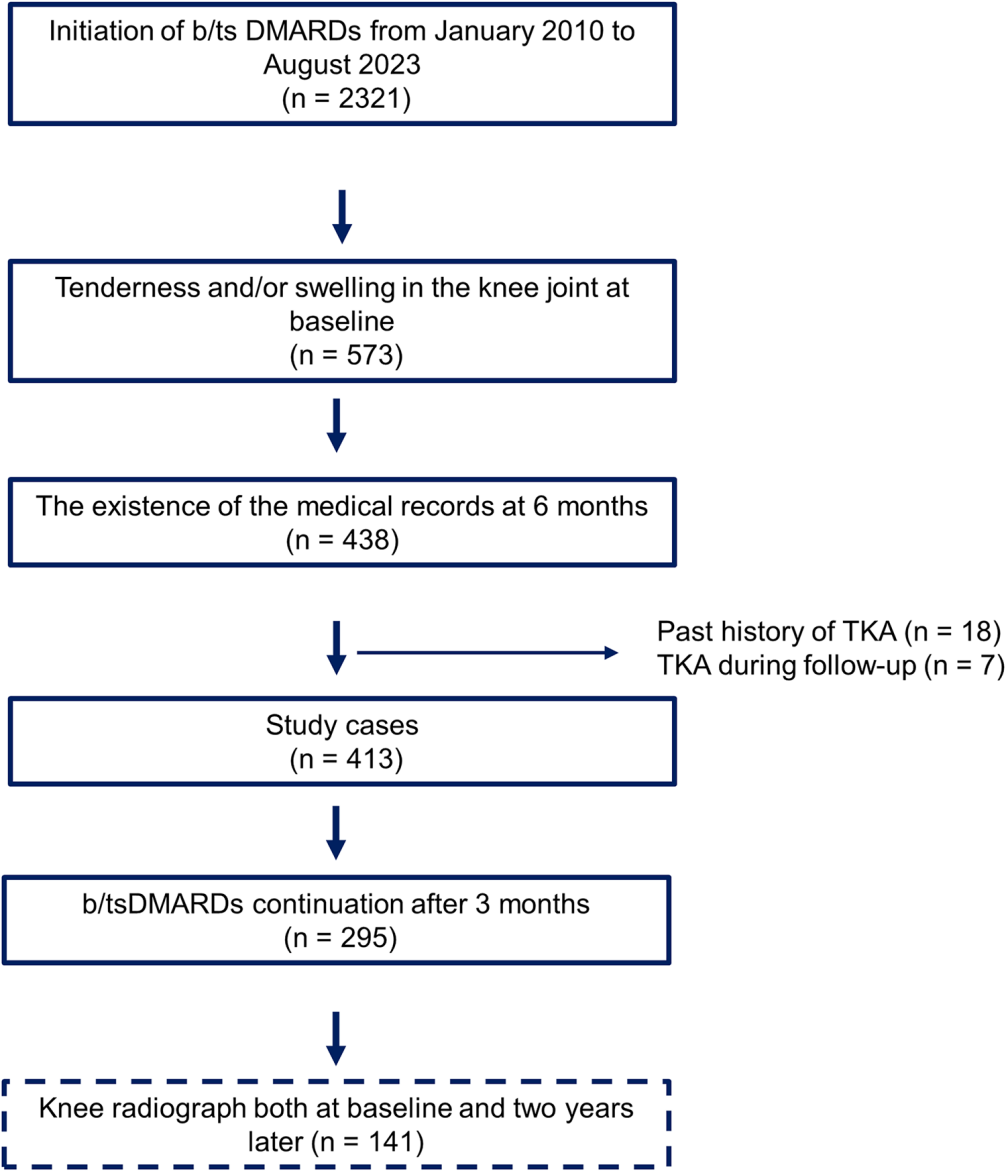
Study flow chart.

From January 2010 to August 2023, a total of 2,321 patients initiated treatment with b/tsDMARDs (Fig. 1). Among them, 573 had knee tenderness and/or swelling at the time of initiating b/tsDMARDs (considered as the baseline). 6 months later, 438 individuals returned for a follow-up, and swelling and/or tender joints were recorded. We excluded patients who had total knee arthroplasty on the symptomatic side (*n* = 18) and those who had undergone TKA during the follow-up period (*n* = 7). As a result, 413 patients were included in the study (Supplementary Table S1). Next, we focused on patients who continued b/tsDMARDs for at least 3 months. Consequently, 295 cases were analyzed. The mean age at baseline was 60.9 years, ACPA positivity was 78.3%, RF positivity was 76.6%, females were 85.1%, and the average RA duration was nine years (Table 1).

**Table 1:** Demographics and disease characteristics of patients with knee joint symptoms at the initiation of b/tsDMARDs treatment who continued the medication for 3 months and exhibited recorded knee joint symptoms after 6 months.

	Total	IL-6 inhibitor	TNF inhibitor	CTLA4-Ig	JAK inhibitor	*p* value
Number of cases	295	78	123	35	59	
Age	60.9 (14.3)	58.8 (14.4)	59.6 (15.3)	66.2 (11.0)	61.7 (13.7)	0.01*
Female (%)	85.1	82.1	82.9	91.5	82.9	0.35^‡^
Disease duration (yr)	9.0 (10.6)	8.5 (10.3)	7.4 (10.2)	12.5 (11.1)	10.0 (11.2)	0.04*
Knee tenderness (%)	88.1	83.3	89.4	88.1	94.3	0.36^‡^
Knee swelling (%)	66.8	73.1	69.9	62.7	48.6	0.05^‡^
TJC	5.4 (5.2)	4.7 (4.4)	5.2 (4.9)	5.1 (4.7)	7.6 (7.6)	0.39^†^
SJC	4.4 (4.5)	4.5 (4.6)	4.0 (3.8)	4.9 (4.6)	5.2 (6.3)	0.69^†^
DAS28-ESR	5.0 (1.1)	5.1 (1.1)	5.0 (1.1)	5.1 (1.1)	5.1 (1.3)	0.9^†^
SDAI	22.8 (12.0)	23.6 (11.4)	21.7 (11.1)	22.5 (12.0)	25.5 (15.5)	0.64^†^
CDAI	20.2 (10.7)	19.9 (9.6)	19.4 (10.1)	20.2 (10.8)	23.7 (14.5)	0.55^†^
ESR (mm/h)	49.3 (30.8)	57.2 (36.8)	47.1 (26.5)	46.5 (27.6)	44.3 (34.1)	0.07*
CRP (mg/dL)	2.5 (3.1)	3.5 (3.8)	2.2 (2.7)	2.2 (2.7)	1.7 (3.1)	0.008*
MMP3 (ng/mL)	363.1 (441.4)	437.7 (469.7)	363.2 (442.6)	273.0 (295.1)	340.0 (555.2)	0.23*
ACPA positivity (%)	78.3	77.8	76.3	90.4	67.7	0.07^‡^
RF positivity (%)	76.6	80.5	71.9	87.9	65.7	0.03^‡^
MTX usage (%)	57.3	52.6	69.9	48.6	42.4	0.001^‡^
MTX dose (mg/week)	8.7 (3.3)	9.4 (3.2)	8.7 (3.3)	6.9 (3.3)	9.3 (3.5)	0.01^†^
Glucocorticoid usage (%)	38	43.6	30.9	42.9	42.4	0.21^‡^
Glucocorticoid dose (mg/day)	5.4 (6.0)	5.4 (2.9)	5.3 (2.5)	4.2 (3.5)	8.3 (14.6)	0.17^†^
1st b/ts DMARDs (%)	60.7	47.4	74.8	69.5	25.7	< 0.001^‡^

Values either represent mean ± standard deviation or percentage.

TJC, tender joint count; SJC, swelling joint count; DAS28-ESR, disease activity score 28-joint count with erythrocyte sedimentation rate; CDAI, clinical disease activity index; SDAI, simplified disease activity index; ESR, erythrocyte sedimentation rate; CRP, C-reactive protein; MMP3, matrix metalloproteinase-3; ACPA, anticitrullinated protein/peptide antibodies; RF, rheumatoid factor; MTX, methotrexate; b/tsDMARDs, biological/targeted synthetic disease-modifying antirheumatic drugs.

*One-way ANOVA; †, Wilcoxon/Kruskal–Wallis test; ‡, Pearson’s chi-square test.

**Figure 2:**
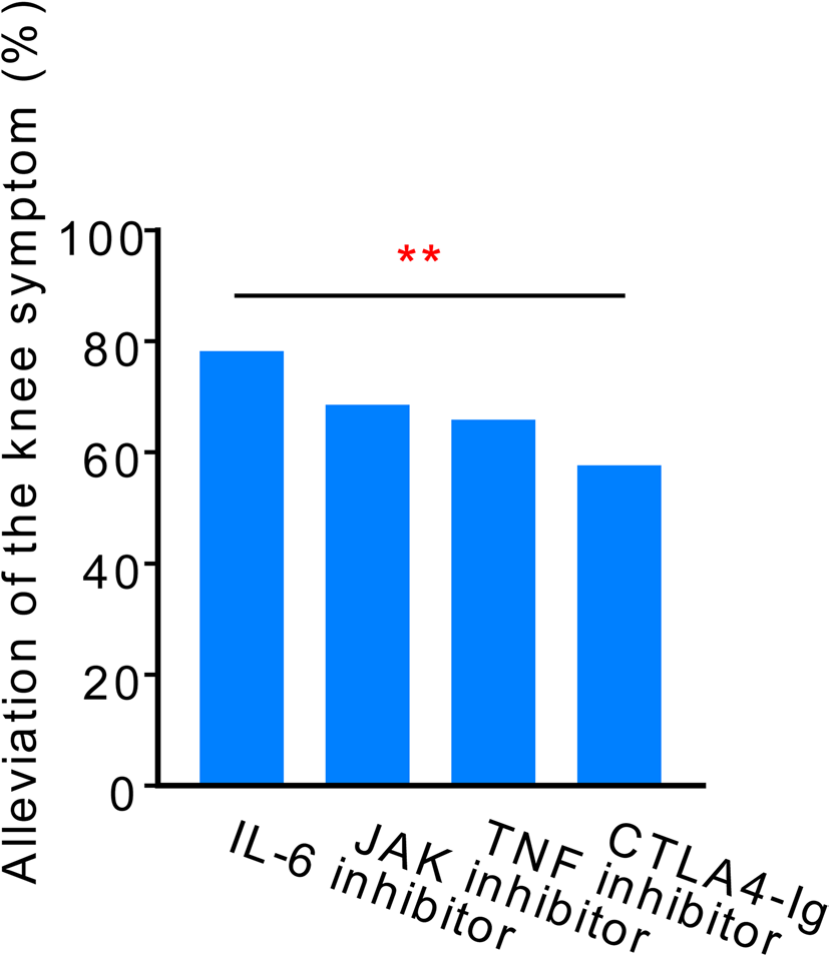
Rates of knee joint symptom alleviation after 6 months of treatment, categorized by each drug’s mode of action, in patients with knee joint symptoms at the initiation of biological/targeted synthetic disease-modifying antirheumatic drugs (b/tsDMARDs) treatment who continued therapy for 3 months. IL-6, interleukin 6; JAK, Janus kinase; CTLA4-Ig, cytotoxic T lymphocyte-associated antigen-4-Ig; TNF, tumor necrosis factor. ** *p* < 0.01 by Cochran-Armitage trend test.

The alleviation of symptoms after 6 months in patients who continued b/tsDMARDs for 3 months was 78.2% for IL-6 inhibitors, 68.6% for JAK inhibitors, 65.9% for TNF inhibitors, and 57.6% for CTLA4-Ig (*p* < 0.01, Fig. 2). The rates of knee joint symptom relief after 3 months were 66.3% for IL-6 inhibitors, 51.2% for JAK inhibitors, 61.4% for TNF inhibitors, and 55.6% for CTLA4-Ig, without statistical significance (*p* = 0.32, Supplementary Fig. S1a). Significant differences were observed in the proportion of improvement in knee joint symptoms between 3 and 6 months for all b/tsDMARDs. Notably, further improvement in knee joint symptoms from 3 to 6 months was noted for both IL-6 and JAK inhibitors (Supplementary Fig. S1b).

**Figure 3:**
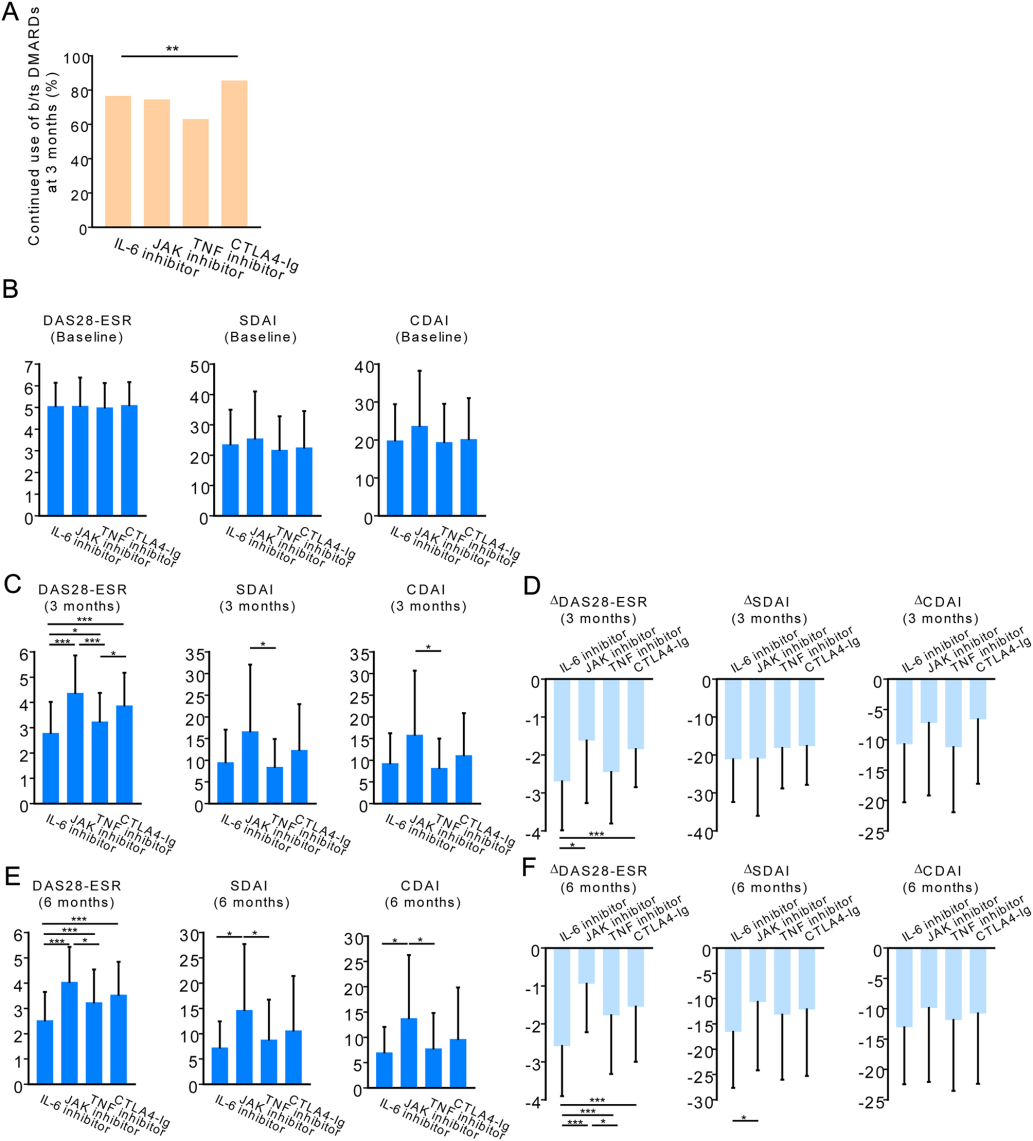
Continuation rates and disease activity for each drug. (**a**) The proportion of patients with knee joint symptoms at the initiation of b/tsDMARDs treatment who continued therapy for 3 months. **; *p* < 0.01, by Chi-square test (**b**) Baseline disease activity (**c**) Disease activity after 3 months of treatment. (**d**) The difference in disease activity from baseline after 3 months of treatment. (**e**) Disease activity after 6 months of treatment. (**f**) The difference in disease activity from baseline after 6 months of treatment. *; *p* < 0.05, **; *p* < 0.01, ***; *p* < 0.001 by Steel–Dwass test. DAS28-ESR, disease activity score 28-joint count with erythrocyte sedimentation rate; CDAI, clinical disease activity index; SDAI, simplified disease activity index

At 3 months from baseline, the continuation rate was 76.5% for IL-6 inhibitors, 74.5% for JAK inhibitors, 63.1% for TNF inhibitors, and 85.5% for CTLA4-Ig (*p* < 0.01; Fig. 3a).

In patients who continued b/tsDMARDs after 3 months, disease activity (DAS28-ESR, disease activity score 28-joint count with erythrocyte sedimentation rate [DAS28-ESR], clinical disease activity index [CDAI], and simplified disease activity index [SDAI]) improved at 3 and 6 months compared with baseline (Fig. 3b–f). Although differences in the values of DAS28-ESR, CDAI, and SDAI at 3 and 6 months, as well as differences from the baseline, were observed among these drugs, IL-6 inhibitors were not inferior to the other drugs in any of these indicators.

**Figure 4:**
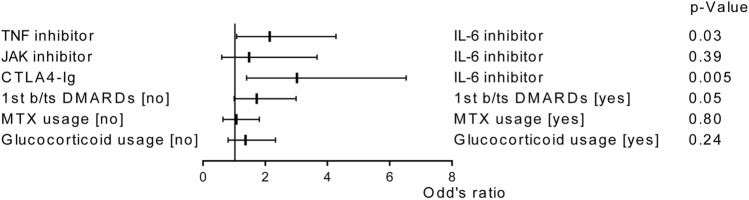
Multivariate logistic regression analysis of knee joint symptom alleviation after 6 months of treatment. MTX, methotrexate.

When investigating treatments associated with the alleviation of knee joint symptoms in patients who continued b/tsDMARDs therapy after 3 months, the utilization of IL-6 inhibitors emerged as a significant contributing factor, outperforming TNF inhibitors in resolving knee joint symptoms (Fig. 4, odds ratio [OR] 2.1, 95% confidence interval [CI] 1.1–4.3, *p* = 0.03) or CTLA4-Ig (OR 3.0, 95% CI 1.4–6.5, *p* = 0.005). The OR for the alleviation of knee joint symptoms when using b/tsDMARDs as the first-line treatment was 1.72 (95% CI 0.99–2.99, *p* = 0.05). Methotrexate (MTX) or glucocorticoid use was not found to be a significant risk factor.

### Factors contributing to the inhibition of knee joint destruction

Next, we focused on 141 patients who continued b/tsDMARD therapy for 3 months and underwent knee joint radiography at baseline and two years later. At baseline, there were no significant differences in disease activity as measured by DAS28-ESR, SDAI, and CDAI (Supplementary Table S2). After 3 months, the DAS28-ESR was higher in patients using JAK inhibitors and CTLA4-Ig, at 4.0 and 4.1, respectively, compared to 3.0 in those using IL-6 inhibitors. The use of MTX was lower in the CTLA4-Ig users; however, this difference was not statistically significant. Additionally, the use rate of JAK inhibitors as first-line b/tsDMARDs was lower.

**Figure 5:**
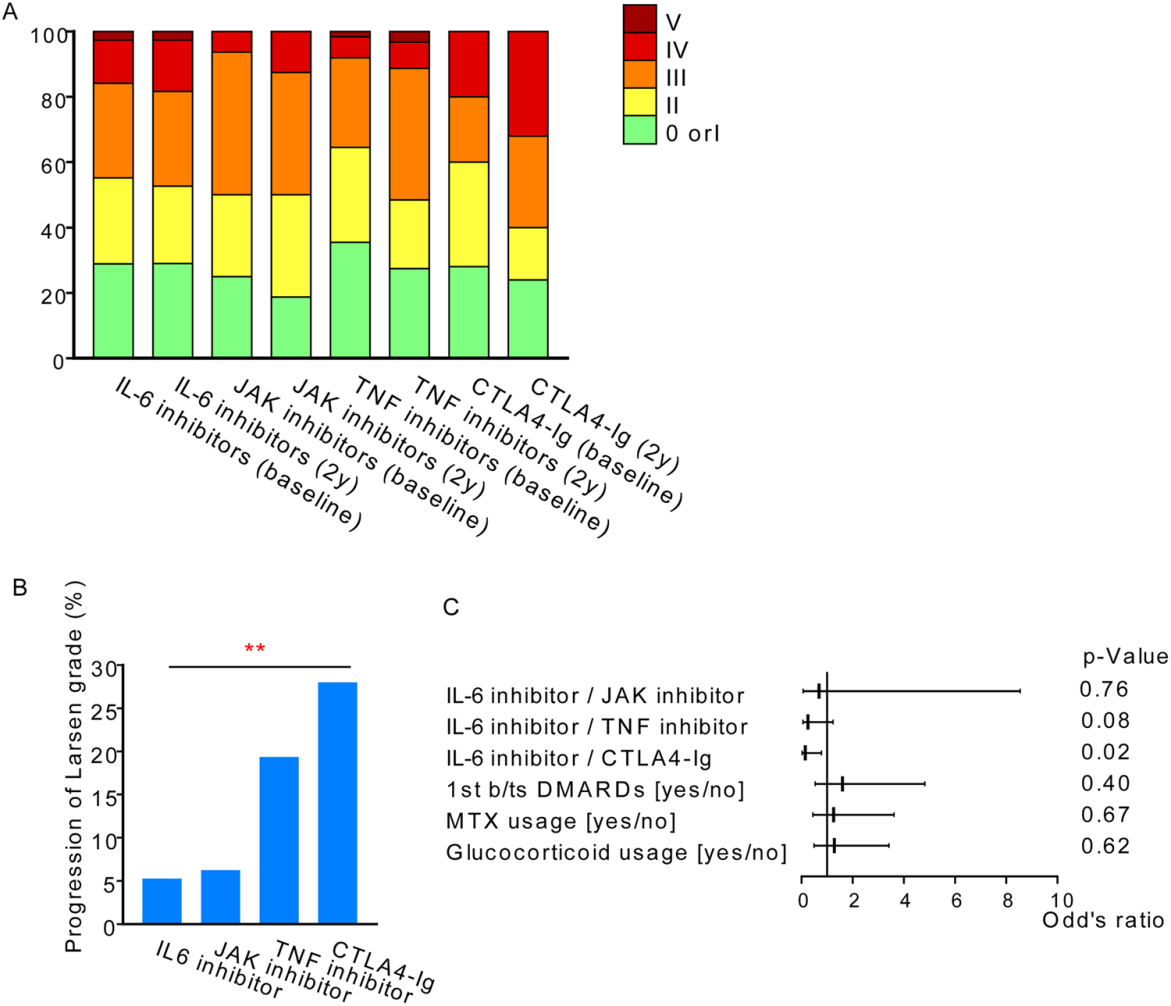
Radiographic evaluation of the knee. (**a**) X-ray image of the knee at baseline and two years, assessed by the Larsen grade. (**b**) Percentage of patients with progression in Larsen grade on X-rays from baseline to two years, excluding progression from grade 0 to I. **; *p* < 0.01 by Cochran-Armitage trend test. (**c**) Factors contributing to the progression of Larsen grade on X-rays from baseline to two years, by multivariate logistic regression analysis.

At baseline, there were no significant differences in the Larsen grade among the various drugs (Fig. 5a). Over two years, excluding the progression from grade 0 to grade I, aggravation of the Larsen grade was observed in 5.2% of patients treated with IL-6 inhibitors, 6.3% with JAK inhibitors, 19.4% with TNF inhibitors, and 28% with CTLA4-Ig (Fig. 5b , *p* < 0.01).

Concordantly, when considering the use of MTX, glucocorticoids, the use of b/tsDMARDs as a first-line treatment, and the mode of action as covariates, the use of IL-6 inhibitors emerged as a protective factor against the worsening of knee joints compared to CTLA4-Ig (OR 0.14, 95% CI 0.02–0.78, *p* = 0.02, Fig. 5c).

## Discussion

In this study, we analyzed the factors influencing the alleviation of knee joint symptoms in patients with RA treated with b/tsDMARDs. The use of IL-6 inhibitors was associated with the resolution of knee joint symptoms compared to the use of TNF inhibitors or CTLA4-Ig. It was also associated with the inhibition of knee joint destruction in comparison to CTLA4-Ig.

It is difficult to completely distinguish between symptoms associated with active synovitis and those associated with structural destruction of the knee joint. Active synovitis should be treated with DMARDs, and structural destruction should be resolved with joint replacement surgery. There was a discrepancy between knee pain and radiographic changes^15^. Although patients with knee synovitis have high serum C-reactive protein (CRP) levels^16^, mild synovitis does not increase serum markers such as CRP and matrix metalloproteinase 3 (MMP3). Testing joint fluid and performing joint ultrasound examinations can also aid in diagnosis^17^. However, it is challenging at times to distinguish joint inflammation symptoms from those caused by structural destruction. Occasionally, an overlap exists. Distinguishing what proportion of these factors contributes to the symptoms is difficult. Therefore, we first performed an analysis that included all knee symptoms and then performed the analysis in several models to exclude non-inflammatory joint symptoms.

Limited information is available on the large joints involvement (LJI) in patients with RA. In a post-hoc analysis of the U-Act-Early trial in 317 patients with very early RA, Rubbert-Roth et al. reported that patients with LJI at baseline had significantly more disease activity and functional disability at baseline^18^. Additionally, while the disease activity score was similar after 2 years of follow-up compared with patients without LJI, the difference in functional disability remained. Furthermore, patients with LJI had a lower chance of achieving drug-free remission. Shirasugi et al. found the similar findings. They showed that CDAI in patients with LJI was higher than in those without LJI. CDAI also remained higher over time in patients with LJI^19^. Another report demonstrated that arthritis of large joints—the knee in particular—was associated with higher CRP levels at presentation with RA and a more destructive disease course^16^.

In a study of 42 patients, Seki et al. reported that after treatment with TNF inhibitors, including IFX and ETN, the radiographic progression of joint damage was inhibited in most grade 0–II weight-bearing joints, but was prominent in those with pre-existing damage (grade III/IV), even in patients responding well to therapy^7^. In a study of 92 patients, Nakajima et al. found progressive damage, defined by the Larsen grade, in 8.7% of the patients. The health assessment questionnaire-disability index score was also associated with the radiographic progression of large joint damage. They concluded that Larsen grade should not exceed three at the start of bDMARD treatment to avoid radiographic progression^20,21^. These data suggest that patients with LJI, including the knee, may have a higher disease activity and a different disease course compared to those without LJI. Additionally, once joint destruction progresses, it may be difficult to control the destruction.

Reports on the effective DMARDs treatment on large joints are scarce. Most studies investigated knee joint damage in patients treated with TNF inhibitors. However, Maeda et al. reported that IL-6 inhibitors were more effective than non-IL-6 inhibitors in improving knee joint symptoms after 3 months^6^. However, reports were limited to symptom alleviation and did not mention any radiographic progression. To the best of our knowledge, our study is the first to demonstrate the distinct contributions of b/tsDMARDs to the radiographic destruction of large joints.The mechanisms through which IL-6 inhibitors alleviate knee joint symptoms remain unknown. Recently, an IL-6 inhibitor was associated with improved disproportionate articular pain, which was more severe than expected based on the amount of joint swelling^22^. Although the detailed mechanism is unknown, the pleiotropic effects of IL-6, especially the expression of gp130 throughout the nervous system, may contribute to pathological pain^23^. Moreover, IL-6 may affect RA-associated fatigue and mood disorders^23^. Treatment with IL-6 inhibitors can positively affect these symptoms and exert substantial anti-inflammatory effects on synovitis.

The limitations of this study include its retrospective design. The extent of structural joint damage and synovitis in the knee may not have been the same across all groups. Concomitant medications may also not be equivalent. Furthermore, b/tsDMARDs selection may have been influenced by factors other than the presence or absence of knee joint symptoms. However, the fact that IL-6 users have higher baseline levels of CRP, ESR, and MMP3 compared to CTLA4-Ig users, along with similar rates of MTX and glucocorticoid usage, and a lower percentage of patients using these as their first b/tsDMARDs, does not detract from the value of this study's results when comparing the effects of IL-6 inhibitors to those of CTLA4-Ig.

Additionally, knee joint symptoms were analyzed without discriminating between inflammatory synovitis and non-inflammatory conditions such as joint deformity and osteoarthritis. Moreover, the study included patients treated with a focus on controlling conditions other than knee joint symptoms. Furthermore, radiography was not performed for all patients, and an accurate radiographic evaluation of the performance of each drug was not possible. Further study, such as a randomized controlled trial focusing on knee, is required to accurately compare the effects of drugs on knee joint symptoms while adjusting for confounding factors.

To conclude, in patients presenting knee joint symptoms at the initiation of b/tsDMARDs treatment and continuing the therapy for 3 months, the utilization of IL-6 inhibitors emerged as a significant factor in the alleviation of knee joint symptoms, surpassing the impact of TNF inhibitors or CTLA4-Ig. Additionally, radiography revealed that in contrast to CTLA4-Ig the use of IL-6 inhibitors was associated with inhibiting the progression of knee joint destruction after two years of treatment initiation. Our findings will be useful for future decision making on the use of b/tsDMARDs in RA patients with knee joint involvement.

## Supplementary Information

Supplementary Information.

## Data Availability

The datasets used and/or analyzed in the current study are available from the corresponding author upon reasonable request.

## References

[1] Smolen, J. S. *et al.* Rheumatoid arthritis. *Nat. Rev. Dis. Primers* **4**, 18001. 10.1038/nrdp.2018.1 (2018). 29417936 10.1038/nrdp.2018.1

[2] Kawahito, Y. *et al.* Drug treatment algorithm and recommendations from the 2020 update of the Japan college of rheumatology clinical practice guidelines for the management of rheumatoid arthritis-secondary publication. *Mod. Rheumatol.* **33**, 21–35. 10.1093/mr/roac017 (2023). 35297492 10.1093/mr/roac017

[3] Smolen, J. S. *et al.* EULAR recommendations for the management of rheumatoid arthritis with synthetic and biological disease-modifying antirheumatic drugs: 2022 update. *Ann. Rheum. Dis.* **82**, 3–18. 10.1136/ard-2022-223356 (2023). 36357155 10.1136/ard-2022-223356

[4] Matsushita, I., Motomura, H., Seki, E. & Kimura, T. Radiographic changes and factors associated with subsequent progression of damage in weight-bearing joints of patients with rheumatoid arthritis under TNF-blocking therapies-3-year observational study. *Mod. Rheumatol.* **27**, 570–575. 10.1080/14397595.2016.1227235 (2017). 27589926 10.1080/14397595.2016.1227235

[5] van der Heijde, D. M. Plain X-rays in rheumatoid arthritis: Overview of scoring methods, their reliability and applicability. *Baillieres Clin. Rheumatol.* **10**, 435–453. 10.1016/s0950-3579(96)80043-4 (1996). 8876953 10.1016/s0950-3579(96)80043-4

[6] Maeda, Y. *et al.* Comparison of efficacy between anti-IL-6 receptor antibody and other biological disease-modifying antirheumatic drugs in the patients with rheumatoid arthritis who have knee joint involvement: The ANSWER cohort, retrospective study. *Rheumatol. Int.* **41**, 1233–1241. 10.1007/s00296-021-04862-y (2021). 33903963 10.1007/s00296-021-04862-y

[7] Seki, E. *et al.* Radiographic progression in weight-bearing joints of patients with rheumatoid arthritis after TNF-blocking therapies. *Clin. Rheumatol.* **28**, 453–460. 10.1007/s10067-008-1076-9 (2009). 19104753 10.1007/s10067-008-1076-9

[8] Murata, K. *et al.* Fluctuation in anti-cyclic citrullinated protein antibody level predicts relapse from remission in rheumatoid arthritis: KURAMA cohort. *Arthritis Res. Ther.* **22**, 268. 10.1186/s13075-020-02366-x (2020). 33183344 10.1186/s13075-020-02366-x PMC7664066

[9] Murata, K. *et al.* Elderly onset of early rheumatoid arthritis is a risk factor for bone erosions, refractory to treatment: KURAMA cohort. *Int. J. Rheum. Dis.* **22**, 1084–1093. 10.1111/1756-185x.13428 (2019). 30415498 10.1111/1756-185X.13428

[10] Watanabe, R. *et al.* Prevalence and predictive factors of difficult-to-treat rheumatoid arthritis: The KURAMA cohort. *Immunol. Med.* **45**, 35–44. 10.1080/25785826.2021.1928383 (2022). 34033729 10.1080/25785826.2021.1928383

[11] Arnett, F. C. *et al.* The American rheumatism association 1987 revised criteria for the classification of rheumatoid arthritis. *Arthritis Rheum.* **31**, 315–324. 10.1002/art.1780310302 (1988). 3358796 10.1002/art.1780310302

[12] Aletaha, D. *et al.* 2010 Rheumatoid arthritis classification criteria: An American college of rheumatology/European league against rheumatism collaborative initiative. *Arthritis Rheum.* **62**, 2569–2581. 10.1002/art.27584 (2010). 20872595 10.1002/art.27584

[13] Ebina, K. *et al.* Drug retention of secondary biologics or JAK inhibitors after tocilizumab or abatacept failure as first biologics in patients with rheumatoid arthritis—the ANSWER cohort study. *Clin. Rheumatol.* **39**, 2563–2572. 10.1007/s10067-020-05015-5 (2020). 32162152 10.1007/s10067-020-05015-5

[14] Larsen, A., Dale, K. & Eek, M. Radiographic evaluation of rheumatoid arthritis and related conditions by standard reference films. *Acta Radiol. Diagn.* **18**, 481–491. 10.1177/028418517701800415 (1977). 10.1177/028418517701800415 920239

[15] Fujii, T. *et al.* Disability due to knee pain and somatising tendency in Japanese adults. *BMC Musculoskelet. Disord.* **19**, 23. 10.1186/s12891-018-1940-y (2018). 29351756 10.1186/s12891-018-1940-y PMC5775591

[16] Linn-Rasker, S. P., van der Helm-van Mil, A. H., Breedveld, F. C. & Huizinga, T. W. Arthritis of the large joints—in particular, the knee—at first presentation is predictive for a high level of radiological destruction of the small joints in rheumatoid arthritis. *Ann. Rheum. Dis.* **66**, 646–650. 10.1136/ard.2006.066704 (2007). 17142384 10.1136/ard.2006.066704 PMC1954616

[17] Najm, A. *et al.* Knee joint synovitis: study of correlations and diagnostic performances of ultrasonography compared with histopathology. *RMD Open* **4**, e000616. 10.1136/rmdopen-2017-000616 (2018). 29531789 10.1136/rmdopen-2017-000616 PMC5845411

[18] Rubbert-Roth, A., Jacobs, J. W. G., Bijlsma, J. W. J. & Welsing, P. M. J. A disconnect between disease activity and functional ability already in patients with early rheumatoid arthritis, depending on large joint involvement. *Ann. Rheum. Dis.* **77**, 1085–1086. 10.1136/annrheumdis-2017-211485 (2018). 28646081 10.1136/annrheumdis-2017-211485

[19] Shirasugi, I. *et al.* Association of large joint involvement at the start of biological disease-modifying antirheumatic drugs and Janus kinase inhibitors with disease activity and drug retention in patients with rheumatoid arthritis: The ANSWER cohort study. *Int. J. Rheum. Dis.* **27**, e15097. 10.1111/1756-185x.15097 (2024). 38439176 10.1111/1756-185X.15097

[20] Nakajima, A. *et al.* Predictive factors for radiographic progression of large joint damage in patients with rheumatoid arthritis treated with biological disease-modifying antirheumatic drugs (bDMARDs): Results of 3 to 4 years of follow-up. *Mod. Rheumatol.* **29**, 903–909. 10.1080/14397595.2018.1532544 (2019). 30285585 10.1080/14397595.2018.1532544

[21] Nakajima, A. *et al.* Health assessment questionnaire-disability index (HAQ-DI) score at the start of biological disease-modifying antirheumatic drug (bDMARD) therapy is associated with radiographic progression of large joint damage in patients with rheumatoid arthritis. *Mod. Rheumatol.* **27**, 967–972. 10.1080/14397595.2017.1294302 (2017). 28271947 10.1080/14397595.2017.1294302

[22] Choy, E. *et al.* Disproportionate articular pain is a frequent phenomenon in rheumatoid arthritis and responds to treatment with sarilumab. *Rheumatology*10.1093/rheumatology/keac659 (2022). 36413080 10.1093/rheumatology/keac659 PMC10321097

[23] Choy, E. H. S. & Calabrese, L. H. Neuroendocrine and neurophysiological effects of interleukin 6 in rheumatoid arthritis. *Rheumatology* **57**, 1885–1895. 10.1093/rheumatology/kex391 (2018). 29186541 10.1093/rheumatology/kex391 PMC6199533

