# Author Correction: Effects of IL-6, JAK, TNF inhibitors, and CTLA4-Ig on knee symptoms in patients with rheumatoid arthritis

**DOI:** 10.1038/s41598-024-81223-2

**Published:** 2024-12-17

**Authors:** Koichi Murata, Ryuji Uozumi, Takayuki Fujii, Akira Onishi, Kosaku Murakami, Hideo Onizawa, Masao Tanaka, Akio Morinobu, Shuichi Matsuda

**Affiliations:** 1 Department of Advanced Medicine for Rheumatic Diseases, Kyoto University Graduate School of Medicine, 54 Shogoin-Kawahara-cho, Sakyo, Kyoto 606-8507 Japan; 2 Department of Orthopaedic Surgery, Kyoto University Graduate School of Medicine, Sakyo, Kyoto Japan; 3 Department of Industrial Engineering and Economics, Tokyo Institute of Technology, Tokyo, 152-8552 Japan; 4 Division of Clinical Immunology and Cancer Immunotherapy, Center for Cancer Immunotherapy and Immunobiology, Kyoto University Graduate School of Medicine, Sakyo, Kyoto 606-8501 Japan; 5 Department of Rheumatology and Clinical Immunology, Kyoto University Graduate School of Medicine, Sakyo, Kyoto 606-8507 Japan

Correction to: *Scientific Reports* 10.1038/s41598-024-66064-3, published online 02 July 2024

The original version of this Article contained an error in Figure 4, where the labels for “JAK inhibitor” and “TNF inhibitor” were reversed. In addition, the values displayed in Figure 5B contained errors. The original Figures 4 and 5 as well as accompanying legends appear below.

**Figure 4:**
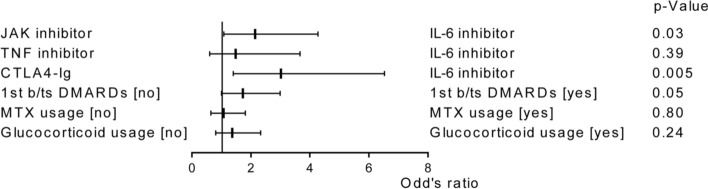
Multivariate logistic regression analysis of knee joint symptom alleviation after 6 months of treatment. MTX, methotrexate.

**Figure 5:**
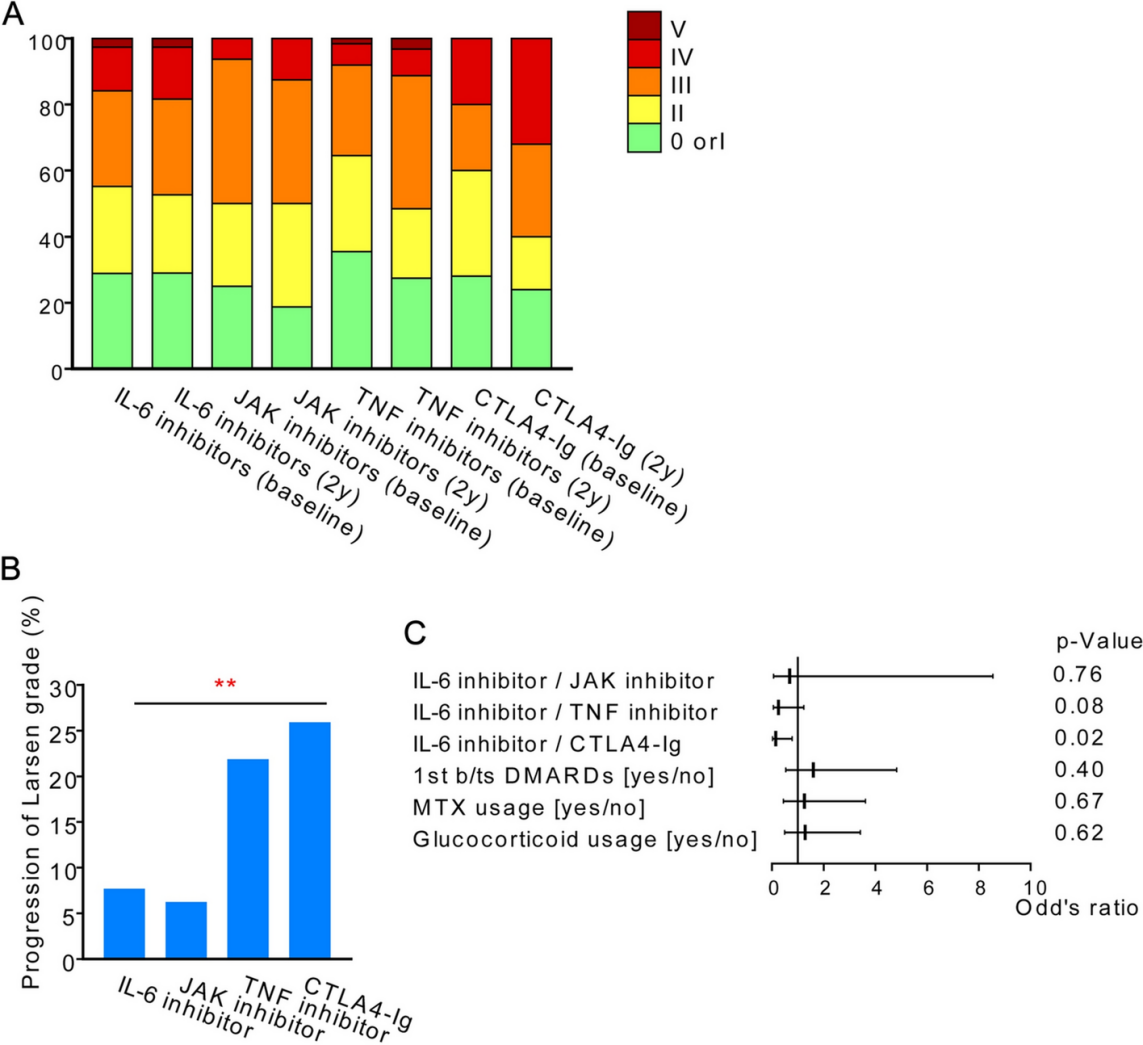
Radiographic evaluation of the knee. (**a**) X-ray image of the knee at baseline and two years, assessed by the Larsen grade. (**b**) Percentage of patients with progression in Larsen grade on X-rays from baseline to two years, excluding progression from grade 0 to I. **; *p* < 0.01 by Cochran-Armitage trend test. (**c**) Factors contributing to the progression of Larsen grade on X-rays from baseline to two years, by multivariate logistic regression analysis.

The original Article has been corrected.

