# Supplementary material for: Effects of IL-6, JAK, TNF inhibitors, and CTLA4-Ig on knee symptoms in patients with rheumatoid arthritis
Source: Sci Rep. 2024 Jul 2;14:15226. doi: 10.1038/s41598-024-66064-3 (PMC11220105; doi:10.1038/s41598-024-66064-3)
Supplement: Supplementary file 1 — Supplementary Information. [file 41598_2024_66064_MOESM1_ESM.docx]

**Supplemental information**

**Effects of IL-6, JAK, TNF Inhibitors, and CTLA4-Ig on Knee Symptoms in Patients with Rheumatoid Arthritis**

Murata et al.

Table of contents

Supplementary Table S1 Page 2

Supplementary Table S2 Page 4

Supplementary Figure S1 Page 6

**Supplementary Table S1.** Demographics and disease characteristics of patients with knee joint symptoms at the initiation of b/tsDMARDs treatment, who had recorded knee joint symptoms after six months.

|  | Total | IL-6 inhibitor | TNF  inhibitor | CTLA4-Ig | JAK inhibitor | p-Value |
| --- | --- | --- | --- | --- | --- | --- |
| Number of cases | 413 | 102 | 195 | 69 | 47 |  |
| Age | 59.9 (14.7) | 59.9 (14.4) | 58.0 (15.5) | 64.1 (13.1) | 61.2 (12.6) | 0.03^*^ |
| Female (%) | 82.6 | 82.4 | 79.5 | 89.9 | 85.1 | 0.26^‡^ |
| Knee tenderness (%) | 88.4 | 85.3 | 88.7 | 88.4 | 93.6 | 0.52^‡^ |
| Knee swelling (%) | 66.6 | 72.6 | 70.3 | 62.3 | 44.7 | 0.003^‡^ |
| TJC | 5.8 (5.6) | 5.2 (5.1) | 5.7 (5.4) | 5.1 (4.6) | 8.6 (7.8) | 0.08^†^ |
| SJC | 4.6 (4.6) | 4.3 (4.3) | 4.6 (4.5) | 4.7 (4.3) | 4.8 (5.8) | 0.74^†^ |
| DAS28-ESR | 5.1 (1.2) | 5.2 (1.1) | 5.1 (1.2) | 5.0 (1.1) | 5.1 (1.3) | 0.8^†^ |
| SDAI | 23.6 (12.1) | 24.0 (11.4) | 23.3 (12.2) | 21.9 (11.5) | 25.8 (14.1) | 0.51^†^ |
| CDAI | 21.0 (10.9) | 20.5 (9.8) | 20.8 (11.0) | 19.7 (10.3) | 24.4 (13.3) | 0.3^†^ |
| ESR (mm/hr) | 49.0 (31.6) | 58.1 (35.9) | 47.6 (29.1) | 45.7 (28.8) | 39.5 (32.3) | 0.004^*^ |
| CRP (mg/dl) | 2.4 (3.1) | 3.3 (3.7) | 2.4 (2.9) | 2.1 (2.7) | 1.4 (2.7) | 0.002^*^ |
| MMP3 (ng/ml) | 357.0 (428.6) | 417.7 (441.6) | 368.4 (441.2) | 281.1 (304.1) | 288.0 (485.8) | 0.18^*^ |
| ACPA positivity (%) | 75.2 | 76.6 | 73.1 | 88.5 | 63 | 0.01^‡^ |
| RF positivity (%) | 75.7 | 82 | 71 | 85.3 | 68.1 | 0.02^‡^ |
| MTX usage (%) | 59.3 | 47.1 | 71.8 | 47.8 | 51.1 | <0.001^‡^ |
| MTX dose (mg/week) | 8.7 (3.4) | 9.1 (3.1) | 8.8 (3.4) | 7.6 (3.6) | 8.9 (3.4) | 0.15^†^ |
| Glucocorticoid usage (%) | 38.3 | 44.1 | 32.3 | 40.6 | 46.8 | 0.11^‡^ |
| Glucocorticoid dose (mg/day) | 5.0 (5.3) | 4.7 (2.9) | 5.1 (3.1) | 4.1 (3.6) | 6.7 (12.2) | 0.22^†^ |
| 1st b/tsDMARDs (%) | 57.9 | 48 | 68.7 | 65.2 | 23.4 | <0.001^‡^ |

Values either represent mean ± standard deviation or percentage.

TJC, tender joint count; SJC, swelling joint count; DAS28-ESR, disease activity score 28-joint count with erythrocyte sedimentation rate; CDAI, clinical disease activity index; SDAI, simplified disease activity index; ESR, erythrocyte sedimentation rate; CRP, C-reactive protein; MMP3, matrix metalloproteinase-3; ACPA, anti-citrullinated protein/peptide antibodies; RF, rheumatoid factor; MTX, methotrexate; b/tsDMARDs, biological/targeted synthetic disease modifying antirheumatic drugs.

*, One-way ANOVA; †, Wilcoxon/Kruskal -Wallis test; ‡, Pearson's chi-squared test

**Supplementary Table S2.** Demographics and disease characteristics of patients with knee joint symptoms at the initiation of b/tsDMARDs treatment, who continued the medication for three months and had X-ray at baseline and after two years.

|  | IL-6 inhibitor | JAK  inhibitor | TNF  inhibitor | CTLA4-Ig | p-Value |
| --- | --- | --- | --- | --- | --- |
| Number of cases | 38 | 16 | 62 | 25 |  |
| Age | 60.5 (12.0) | 67.8 (11.2) | 63.7 (11.7) | 62.2 (10.3) | 0.2^*^ |
| Female (%) | 92.1 | 93.8 | 83.9 | 88 | 0.53^‡^ |
| Knee tenderness (%) | 89.5 | 87.5 | 88.7 | 92 | 0.97^‡^ |
| Knee swelling (%) | 76.3 | 75 | 72.6 | 60 | 0.53^‡^ |
| TJC | 5.4 (4.5) | 4.9 (4.1) | 5.4 (6.0) | 6.3 (4.8) | 0.36^†^ |
| SJC | 4.8 (5.0) | 4.2 (4.3) | 3.8 (3.3) | 5.1 (4.8) | 0.71^†^ |
| DAS28-ESR (baseline) | 5.1 (1.3) | 4.8 (1.2) | 5.1 (1.1) | 5.3 (1.2) | 0.67^†^ |
| SDAI (baseline) | 25.6 (12.7) | 20.7 (10.6) | 22.2 (12.3) | 23.4 (13.5) | 0.46^†^ |
| CDAI (baseline) | 22.1 (10.7) | 18.7 (9.1) | 19.3 (10.9) | 21.5 (12.5) | 0.53^†^ |
| ESR (mm/hr, baseline) | 51.5 (34.2) | 43.5 (31.4) | 48.0 (28.7) | 46.7 (30.6) | 0.83^*^ |
| CRP (mg/dl, baseline) | 3.2 (3.4) | 1.9 (4.0) | 2.4 (3.1) | 1.8 (1.9) | 0.28^*^ |
| MMP3 (ng/ml, baseline)) | 357.5 (322.0) | 495.3 (853.6) | 357.6 (412.7) | 237.2 (176.9) | 0.39^*^ |
| Knee symptom (%, 3 months) | 47.2 | 53.9 | 49.2 | 50 | 0.98^‡^ |
| DAS28-ESR (3 months) | 3.0 (1.3) | 4.0 (1.1) | 3.4 (1.1) | 4.1 (1.6) | 0.0086^†^ |
| SDAI (3 months) | 12.0 (8.5) | 11.9 (8.7) | 9.4 (6.1) | 14.9 (11.8) | 0.3^†^ |
| CDAI (3 months) | 11.4 (7.2) | 10.9 (8.3) | 9.4 (7.3) | 13.8 (11.1) | 0.35^†^ |
| ESR (mm/hr, 3 months) | 15.4 (21.6) | 41.4 (27.2) | 31.3 (26.5) | 39.3 (28.3) | <0.001^*^ |
| CRP (mg/dl, 3 months) | 0.6 (2.1) | 1.1 (2.0) | 0.8 (1.5) | 1.1 (1.2) | 0.63^*^ |
| MMP3 (ng/ml, 3 months) | 155.6 (142.0) | 168.7 (207.5) | 230.3 (298.7) | 237.2 (329.1) | 0.53^*^ |
| Knee symptom (%, 6 months) | 30.3 | 45.5 | 39.3 | 39.8 | 0.41^‡^ |
| DAS28-ESR (6 months) | 2.8 (1.0) | 3.5 (1.1) | 3.4 (1.2) | 3.7 (1.6) | 0.04^†^ |
| SDAI (6 months) | 9.2 (5.2) | 10.6 (8.5) | 9.7 (8.0) | 13.4 (14.2) | 0.95^†^ |
| CDAI (6 months) | 8.9 (5.2) | 10.6 (8.5) | 8.7 (7.3) | 12.8 (13.8) | 0.27^†^ |
| ESR (mm/hr, 6 months) | 14.7 (17.4) | 33.9 (24.6) | 30.5 (25.3) | 31.2 (27.6) | 0.004^*^ |
| CRP (mg/dl, 6 months) | 0.3 (0.7) | 0.7 (1.6) | 0.9 (2.3) | 0.7 (1.0) | 0.31^*^ |
| MMP3 (ng/ml, 6 months) | 122.8 (100.2) | 249.8 (394.0) | 201.0 (247.6) | 194.6 (309.3) | 0.34^*^ |
| ACPA positivity (%) | 78.4 | 35.7 | 76.4 | 91.3 | 0.001^‡^ |
| RF positivity (%) | 78.4 | 56.3 | 77.1 | 95.8 | 0.02^‡^ |
| MTX usage (%, baseline) | 55.3 | 62.5 | 72.6 | 48 | 0.12^‡^ |
| MTX dose (mg/week, baseline) | 8.7 (2.8) | 7.8 (2.9) | 8.8 (3.8) | 6.5 (2.8) | 0.18^†^ |
| Glucocorticoid usage (%, baseline) | 47.4 | 31.3 | 32.3 | 44 | 0.4^‡^ |
| Glucocorticoid dose (mg/day, baseline) | 4.9 (2.8) | 4.6 (2.0) | 5.0 (2.7) | 3.9 (1.8) | 0.72^†^ |
| MTX usage (%, 3 months) | 52.6 | 56.3 | 72.6 | 52 | 0.12^‡^ |
| MTX dose (mg/week, 3 months) | 8.3 (3.5) | 7.8 (2.5) | 9.0 (4.0) | 6.3 (2.8) | 0.14^†^ |
| Glucocorticoid usage (%, 3 months) | 52.6 | 31.3 | 33.9 | 56 | 0.1^‡^ |
| Glucocorticoid dose (mg/day, 3 months) | 4.7 (3.1) | 4.2 (2.5) | 3.9 (1.6) | 4.1 (2.5) | 0.95^†^ |
| 1st b/tsDMARDs (%) | 51.3 | 18.8 | 70.3 | 66.7 | 0.001^‡^ |

*, One-way ANOVA; †, Wilcoxon/Kruskal -Wallis test; ‡, Pearson's chi-squared test

**Supplementary Figure S1** (a) Rates of knee joint symptom alleviation after three months of treatment, categorized by each drug's mode of action, in patients with knee joint symptoms at the initiation of biological/targeted synthetic disease modifying antirheumatic drugs (b/tsDMARDs) treatment, with continuation of drugs for three months. (b) The rates of knee joint symptom alleviation at 3 and 6 months after the initiation of b/tsDMARDs treatment.
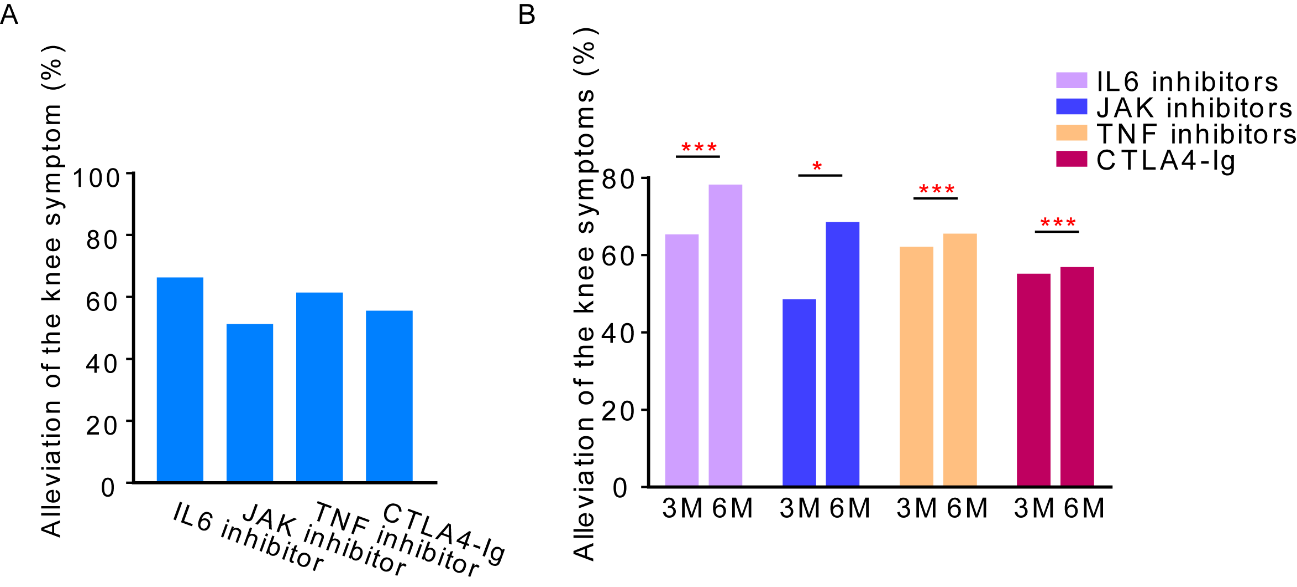


IL-6, interleukin 6; JAK, Janus kinase, CTLA4-Ig, cytotoxic T lymphocyte-associated antigen-4-Ig; TNF, tumor necrosis factor

*; p < 0.05, ***; p < 0.001 by Fisher’s exact test
